# Fasting plasma glucose and serum uric acid levels in a general Chinese population with normal glucose tolerance: A U-shaped curve

**DOI:** 10.1371/journal.pone.0180111

**Published:** 2017-06-28

**Authors:** Yunyang Wang, Jingwei Chi, Kui Che, Ying Chen, Xiaolin Sun, Yangang Wang, Zhongchao Wang

**Affiliations:** 1 Department of Endocrinology & Metabolism, Affiliated Hospital of Qingdao University, Qingdao, Shandong Province, China; 2 Laboratory of Thyroid Disease, Affiliated Hospital of Qingdao University, Qingdao, Shandong Province, China; University of Catanzaro, ITALY

## Abstract

**Objective:**

Although several epidemiological studies assessed the relationship between fasting plasma glucose (FPG) and serum uric acid (SUA) levels, the results were inconsistent. A cross-sectional study was conducted to investigate this relationship in Chinese individuals with normal glucose tolerance.

**Research design and methods:**

A total of 5,726 women and 5,457 men with normal glucose tolerance were enrolled in the study. All subjects underwent a 75-g oral glucose tolerance test. Generalized additive models and two-piecewise linear regression models were applied to assess the relationship.

**Results:**

A U-shaped relationship between FPG and SUA was observed. After adjusting for potential confounders, the inflection points of FPG levels in the curves were 4.6 mmol/L in women and 4.7 mmol/L in men respectively. SUA levels decreased with increasing fasting plasma glucose concentrations before the inflection points (regression coefficient [*β*] = -36.4, *P* < 0.001 for women; *β* = -33.5, *P* < 0.001 for men), then SUA levels increased (*β* = 17.8, *P* < 0.001 for women; *β* = 13.9, *P* < 0.001 for men). Additionally, serum insulin levels were positively associated with FPG and SUA (*P* < 0.05).

**Conclusions:**

A U-shaped relationship between FPG and SUA levels existed in Chinese individuals with normal glucose tolerance. The association is partly mediated through serum insulin levels.

## Introduction

Uric acid, the final oxidation product of purine metabolism in human beings, possesses both antioxidant and pro-oxidant properties [1]. The levels of serum uric acid (SUA) are determined by a balance of production, reabsorption and secretion [2]. Because of the evolutionary loss of hepatic uricase by mutational silencing, uric acid is present at higher levels in human blood than in other mammals [3].

Uric acid has proven emerging roles in various diseases such as gout, renal dysfunction, hypertension, hyperlipidemia, diabetes and obesity [4–7]. Hyperuricemia occurs as a result of the abnormal increased uric acid production and/or the impaired renal uric acid excretion [8]. As a concomitant of metabolic syndrome, hyperuricemia is an independent risk factor of impaired fasting glucose and type 2 diabetes [9, 10]. Clarifying the association between plasma glucose and SUA levels in population with normal glucose tolerance benefits the screening and prevention of diabetes.

A noticeable relationship has been observed between plasma glucose and SUA levels. Interestingly, the relationship between the two factors does not show a simple linear correlation. Studies conducted in diverse ethnic groups showed similar results that fasting plasma glucose (FPG) and SUA exhibited a curvilinear correlation, both in general population and diabetic subjects [11–19]. However, most investigations didn’t exclude individuals with glucose intolerance, leaving the relationship among the subjects with normal glucose tolerance unclear. Furthermore, the inflection point of the curve remains controversial.

In order to study the relationship between FPG and SUA levels in Chinese individuals with normal glucose tolerance, we conducted this study in a Chinese adult population.

## Methods

### Study participants

We conducted a series of investigations from 2004 to 2014 and a stratified, random cluster sampling method was carried out to select a representative sample of the general population aged from 20 to 80 years old in coastal areas of Shandong Province. The sampling process was stratified according to geographic regions (Qingdao, Yantai, Weihai, Rizhao and Dongying), degree of urbanization (cities, county seats and rural townships) and economic development status (based on the gross domestic product for each area). A total of 16,572 people were selected and invited to participate in the study between August 2004 and December 2014; 14,361 individuals (8,123 women and 6,238 men) completed the investigation. The overall response rate was 86.7% (82.8% for men and 89.9% for women). Subjects’ data and blood samples were collected during the investigations. Due to missing data on demographic information or lab measurements, taking diuretic, hyperuricemic or hypouricemic agents, showing signs of malignancy, acute infectious diseases, acute inflammatory diseases and renal failure, 358 individuals were excluded. A standard 75g 2-hour oral glucose tolerance test (OGTT) was administered to all participants. Among 14,003 individuals, 2820 subjects were excluded due to glucose intolerance (FPG level ≥ 6.1 mmol/L, OGTT 2 hour plasma glucose ≥ 7.8 mmol/L) or taking hypoglycemic agents, leaving 11183 subjects with normal glucose tolerance included in the final analysis.

The study protocol was approved by Ethics Committee of Affiliated Hospital Qingdao University, in accordance with the principles of the Declaration of Helsinki. Written informed consent was obtained from all participants. The authors had no access to information that could identify individual participants during or after data collection.

### Biochemical measurements

After an overnight fast (at least 10 hours), blood samples were collected during the investigations. Glycosylated hemoglobin (HbA1c) was measured by high performance liquid chromatography (Bio-Rad Variant Ⅱ HbA1c analyzer, Bio-Rad, Montreal, Quebec, Canada). Serum insulin was measured by Electrochemiluminescence method (Cobas e 601, Roche Diagnosis, Mannheim, Germany). Plasma glucose (glucose oxidase method), serum uric acid (Uricase-PAP/TOOS method), serum total cholesterol (CHOD-PAP method), serum high-density lipoprotein cholesterol (HDL-C) (IRC method), serum low-density lipoprotein cholesterol (LDL-C) (CAT method), serum triglycerides (GPO-PAP method) and serum creatinine (Jaffe reaction) were measured on an analyzer (Hitachi 7600–020, Hitachi, Tokyo, Japan).

The estimated glomerular filtration rate (eGFR) was calculated using the Chronic Kidney Disease Epidemiology (CKD-EPI) equation [20]:

$${eGFR}_{CKD-EPI}=141\times {min(Scr/\kappa ,\;1)}^{\alpha }\times {max(Scr/\kappa ,\;1)}^{-1.209}\times 0.993^{age}\times 1.018\;[if\;female]\times 1.159\;[if\;black]$$

where Scr is serum creatinine expressed in mg/dL and age is expressed in years; κ is 0.7 for females and 0.9 for males; α is–0.329 for females and –0.411 for males; min indicates the minimum of Scr/κ or 1, and max indicates the maximum of Scr/κ or 1.

Homeostasis model assessment for insulin resistance (HOMA-IR) estimates insulin resistance [21].

$$HOMA-IR=\left(\frac{fasting\;insulin\;(\mu IU/mL)\times fasting\;glucose\;(mmol/L)}{22.5}\right)$$

### Statistical analyses

Statistical analyses were conducted using R software program version 3.2.2 (http://www.R-project.org) and SPSS 16.0 (SPSS Inc., Chicago, IL, USA). Data were given as means ± SEM or median (interquartile range). The Kolmogorov–Smirnov *Z* tests were used to determine if the data were normally distributed. Differences between two groups were compared by independent two-tailed Student’s *t* tests for normally distributed data, otherwise by Mann-Whitney *U* tests. We could not assume a linear relationship between FPG and SUA, so the possible non-linear relationship was explored using smoothing splines generated in generalized additive models [22] by R package mgcv. A two-piecewise linear regression model was applied to examine the threshold effect of FPG on uric acid according to the smoothing splines. The inflection point of FPG levels, at which the relationship between FPG and SUA began to reverse, was determined by using trial and error methods, including selection of inflection points along a predefined interval and then choosing the inflection point that gave the maximum likelihood. Furthermore, we performed linear regression analyses to estimate the relationship between FPG and uric acid levels within each stratum FPG with or without adjustment for potential confounders. The Chow tests [23] was used to assess the difference of regression coefficients between strata with R package strucchange. The differences of regression coefficients between models were compared by Student’s *t* tests. Univariate correlations were analyzed by Spearman’s rank correlation tests. A *P* value less than 0.05 (2-tailed) was considered statistically significant.

## Results

### Subject characteristics

Clinical characteristics of the subjects are shown in Table 1. SUA levels in men were significantly higher than in women (348 [106] vs. 263 [85] μmol/L, *P* < 0.001). FPG concentrations in men were also significantly higher than in women (5.2 [0.7] vs. 5.1 [0.7], *P* < 0.001). There was no significant difference in age, body mass index, hemoglobin A1c, total cholesterol, LDL cholesterol, fasting serum insulin, antihypertensive medication and antihyperlipidemic medication between men and women. Waist circumference, hip circumference, waist-hip ratio, systolic blood pressure, diastolic blood pressure, triglycerides, creatinine and eGFR were significantly higher in men than in women, while OGTT 2h plasma glucose and HDL cholesterol were significantly lower in men than in women. Rates of current smoker and everyday drinker were significantly higher in men.

**Table 1:** Clinical and metabolic characteristics of the study population by gender.

	Women	Men	*P* value
**Number of participants**	5726	5457	
**Age (year)**	47 (39, 57)	47 (37, 58)	0.873
**Body mass index (kg/m**^**2**^**)**	24.20 (21.79, 26.84)	24.23 (21.75, 26.67)	0.455
**Waist circumference (cm)**	85 (78, 92)	89 (83, 96)	< 0.001
**Hip circumference (cm)**	99 (94, 104)	100 (95, 105)	< 0.001
**Waist-hip ratio**	0.85 (0.81, 0.90)	0.89 (0.85, 0.93)	< 0.001
**Systolic blood pressure (mmHg)**	121 (110, 140)	130 (120, 140)	< 0.001
**Diastolic blood pressure (mmHg)**	80 (71, 89)	84 (80, 90)	< 0.001
**Fasting plasma glucose (mmol/L)**	5.1 (4.7, 5.4)	5.2 (4.8, 5.5)	< 0.001
**OGTT 2h plasma glucose (mmol/L)**	6.6 (5.9, 7.1)	6.5 (5.8, 7.0)	< 0.001
**Hemoglobin A1c (%)**	5.5 (5.2, 5.7)	5.5 (5.2, 5.7)	0.539
**Serum uric acid (μmol/L)**	263 (224, 309)	348 (298, 404)	< 0.001
**Total cholesterol (mmol/L)**	4.81 (4.24, 5.48)	4.79 (4.22, 5.37)	0.016
**HDL cholesterol (mmol/L)**	1.47 (1.28, 1.68)	1.34 (1.15, 1.58)	< 0.001
**LDL cholesterol (mmol/L)**	2.63 (2.18, 3.20)	2.65 (2.21, 3.18)	0.608
**Triglycerides (mmol/L)**	1.16 (0.82, 1.71)	1.28 (0.89, 2.03)	< 0.001
**Creatinine (μmol/L)**	56.1 (49.7, 63.5)	77.8 (70.0, 86.4)	< 0.001
**eGFR (mL/min/1.73m**^**2**^**)**	104.7 (95.0, 114.1)	99.0 (88.6, 109.5)	< 0.001
**Serum insulin (μIU/mL)**	7.92 (5.63, 10.86)	6.70 (4.18, 10.05)	< 0.001
**HOMA-IR**	1.76 (1.19, 2.48)	1.51 (0.89, 2.33)	< 0.001
**Antihypertensive medication**	653 (11.4%)	649 (11.9%)	0.420
**Antihyperlipidemic medication**	120 (2.1%)	109 (2.0%)	0.714
**Current smoker**	135 (2.4%)	2453 (45.0%)	< 0.001
**Everyday drinker**	71 (1.2%)	1130 (20.7%)	< 0.001

Data are presented as median (interquartile range) for continuous variables or number of subjects (%) for categorical variables. OGTT = oral glucose tolerance test, HDL = high-density lipoprotein, LDL = low-density lipoprotein, eGFR = estimated glomerular filtration rate, HOMA-IR = homeostasis model assessment for insulin resistance.

### Gender-specific univariate regression analyses of the relationship between fasting plasma glucose and serum uric acid

**Fig 1:**
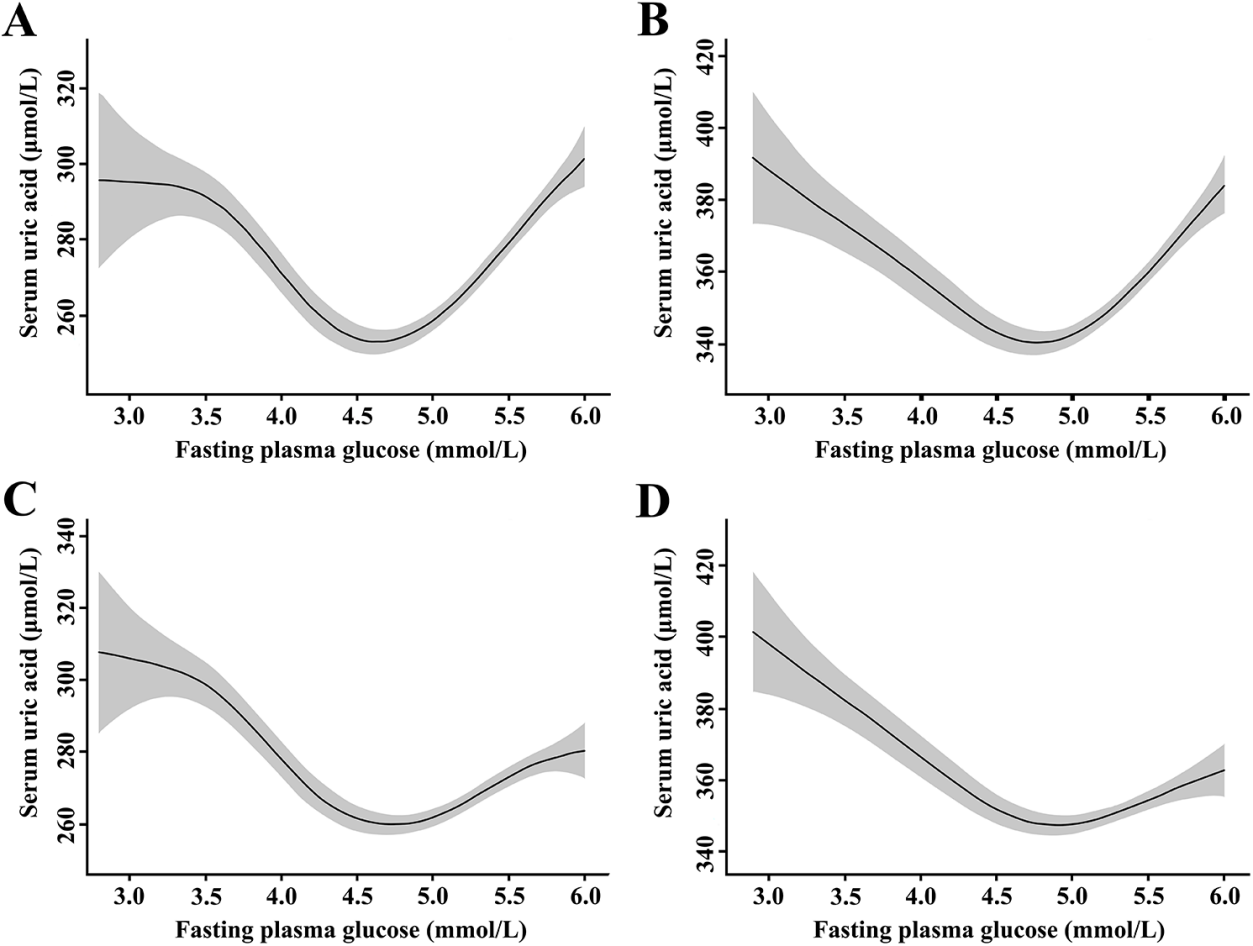
Nonlinear relationship between fasting plasma glucose and serum uric acid. No adjustment in women (A) and in men (B); adjustment for age, body mass index, systolic body pressure, diastolic blood pressure, total cholesterol, triglycerides, drinking status, smoking status, estimated glomerular filtration rate and serum insulin in women (C) and in men (D). The shaded areas indicate 95% confidence intervals.

The relationship between FPG and SUA is shown in Fig 1. The analyses of the relationship were performed after stratifying participants by gender. Smoothing splines suggested a U-shaped relationship between FPG and SUA levels. The SUA levels decreased with increasing FPG levels before the inflection points of 4.6 (women)/4.7 (men) mmol/L, then the SUA levels increased after the inflection points. The regression coefficients were -38.8 (95% confidence interval [CI]: -44.9 to -32.7, *P* < 0.001) for FPG < 4.6 mmol/L while 36.5 (95% CI: 31.9 to 41.0, *P* < 0.001) for FPG ≥ 4.6 mmol/L in women, and the difference between strata was significant (*F* = 27.7, *P* < 0.001). Additionally, the regression coefficients were -35.2 (95% CI: -42.5 to -27.8, *P* < 0.001) for FPG < 4.7 mmol/L while 35.4 (95% CI: 29.2 to 41.6, *P* < 0.001) for FPG ≥ 4.7 mmol/L in men, and the difference between strata was significant (*F* = 27.2, *P* < 0.001) (Table 2).

**Table 2:** Linear regression analyses between fasting plasma glucose and serum uric acid.

				Crude^b^		Model 1^c^		Model 2^d^		Model 3^e^	
Fasting plasma glucose group (mmol/L)	N	Fasting plasma glucose (mmol/L)^a^	Uric acid (μmol/L)^a^	*β* (95% CI)	*P*	*β* (95% CI)	*P*	*β* (95% CI)	*P*	*β* (95% CI)	*P*
Women											
< 4.6	1158	4.1 (3.6, 4.4)	267 (223, 314)	-38.8 (-44.9, -32.7)	< 0.001	-37.8 (-43.9, -31.7)	< 0.001	-36.7 (-42.8, -30.7)	< 0.001	-36.4 (-42.2, -30.6)	< 0.001
≥ 4.6	4568	5.2 (4.9, 5.5)	262 (225, 308)	36.5 (31.9, 41.0)	< 0.001	35.5 (31.0, 40.1)	< 0.001	33.5 (29.0, 38.1)	< 0.001	17.8 (13.2, 22.4)	< 0.001
Difference between strata				75.3 (66.1, 84.4)	< 0.001	73.3 (64.1, 82.5)	< 0.001	70.3 (61.2, 79.4)	< 0.001	54.2 (45.3, 63.0)	< 0.001
Men											
< 4.7	1097	4.2 (3.7, 4.5)	350 (297, 411)	-35.2 (-42.5, -27.8)	< 0.001	-35.5 (-42.9, -28.2)	< 0.001	-34.9 (-42.2, -27.6)	< 0.001	-33.5 (40.6, -26.4)	< 0.001
≥ 4.7	4360	5.3 (5.1, 5.6)	348 (298, 403)	35.4 (29.2, 41.6)	< 0.001	35.8 (29.6, 42.0)	< 0.001	34.8 (28.7, 41.0)	< 0.001	13.9 (7.5, 20.4)	< 0.001
Difference between strata				70.6 (59.0, 82.2)	< 0.001	71.3 (59.7, 82.9)	< 0.001	69.7 (58.2, 81.3)	< 0.001	47.4 (35.9, 58.9)	< 0.001

Linear regression analyses were conducted separately in strata for women and men. *β*, regression coefficient; CI, confidence interval.

^a^Data are presented as median (interquartile range).

^b^No adjustment.

^c^Adjustment for age.

^d^Additional adjustment for body mass index, systolic body pressure, diastolic blood pressure, total cholesterol, triglycerides, drinking status, smoking status and estimated glomerular filtration rate.

^e^Additional adjustment for serum insulin.

### Gender-specific multivariate regression analyses of the relationship between fasting plasma glucose and serum uric acid

In addition to univariate regression analyses, the relationship between FPG and SUA was analyzed after adjusting age, body mass index, systolic body pressure, diastolic blood pressure, total cholesterol, triglycerides, drinking status, smoking status and estimated glomerular filtration rate (Fig 1). The inflection points of FPG were 4.6 (women)/4.7 (men) mmol/L after adjustment, and the adjusted regression coefficients were -36.7 (95% CI: -42.8 to -30.7, *P* < 0.001) for FPG < 4.6 mmol/L while 33.5 (95% CI: 29.0 to 38.1, *P* < 0.001) for FPG ≥ 4.6 mmol/L in women, and the difference between strata was significant (*F* = 26.9, *P* < 0.001). Additionally, the adjusted regression coefficients were -34.9 (95% CI: -42.2 to -27.6, *P* < 0.001) for FPG < 4.7 mmol/L while 34.8 (95% CI: 28.7 to 41.0, *P* < 0.001) for FPG ≥ 4.7 mmol/L in men, and the difference between strata was significant (*F* = 26.9, *P* < 0.001) (Table 2).

Besides glucose metabolism, insulin may participate in regulation of uric acid levels as well. Therefore, the serum insulin level was included as an adjusting factor in the multivariate regression model. Compared with the models without serum insulin, the inflection points for each gender remained constant. The adjusted regression coefficients after the inflection points were 17.8 (95% CI: 13.2 to 22.4, *P* < 0.001) for women and 13.9 (95% CI: 7.5 to 20.4, *P* < 0.001) for men. For women with FPG ≥ 4.6 mmol/L, the difference of the adjusted regression coefficient between model 2 and model 3 was 15.7 (95% CI: 9.3 to 22.1, *P* < 0.001). In addition, for male participants with FPG ≥ 4.7 mmol/L, the difference was 20.9 (95% CI: 12.1 to 29.7, *P* < 0.001).

### Univariate correlation analyses between serum uric acid and glucose homeostasis parameters

**Fig 2:**
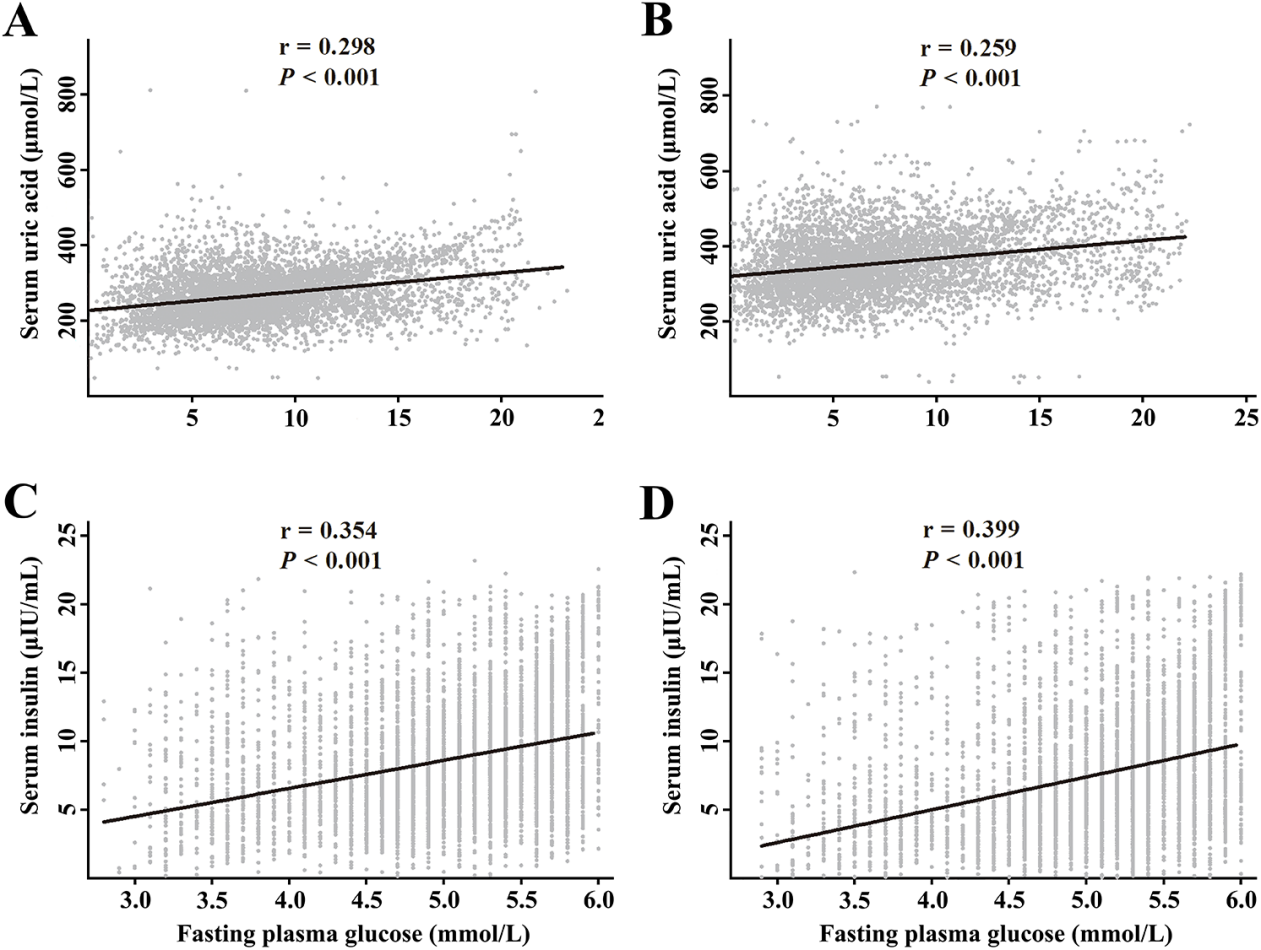
Relationships among serum insulin, fasting plasma glucose, and serum uric acid. Positive relationship between serum insulin and serum uric acid in women (A) and in men (B). Positive relationship between fasting plasma glucose and serum insulin in women (C) and in men (D).

Serum insulin levels showed positive correlations with SUA (*r* = 0.298, *P* < 0.001 for women; *r* = 0.259, *P* < 0.001 for men) and FPG (*r* = 0.354, *P* < 0.001 for women; *r* = 0.399, *P* < 0.001 for men) (Fig 2). It was also found that HOMA-IR was positively associated with SUA (*r* = 0.287, *P* < 0.001 for women; *r* = 0.249, *P* < 0.001 for men), FPG (*r* = 0.533, *P* < 0.001 for women; *r* = 0.531, *P* < 0.001 for men) and serum insulin (*r* = 0.972, *P* < 0.001 for women; *r* = 0.984, *P* < 0.001 for men).

## Discussion

In subjects with normal glucose tolerance, a U-shaped curve was found in the adjusted association between FPG and SUA. The inflection points of FPG levels were 4.6 mmol/L in women and 4.7 mmol/L in men after adjustment. Additionally, serum insulin levels were positively associated with FPG and SUA. Uric acid metabolism is a complex process involving various factors that regulate renal and gut excretion of this compound, so different metabolic pathways may play the predominate role in each segment of the curve. Hence, this nonlinear relationship may indicate the potential interaction between serum glucose and purine metabolism.

In the descending segment of the curve, competition for reabsorption between glucose and uric acid may exert strong influence. Normally, renal elimination of uric acid accounts for more than 70% of total uric acid excretion from the body [24]. The exact mechanisms have not been known, however, multiple studies have led to a proposed four-component model that includes glomerular filtration, reabsorption, secretion and post-secretory reabsorption [25]. Almost 100% of circulating uric acid is filtered at the glomerulus into the renal tubule, with over 90% of the filtered load reabsorbed [26]. Uric acid reabsorption primarily occurs in the proximal tubular by transporters which could exchange intracellular anions, and post-secretory reabsorption takes place in the distal end of the proximal tubular [25, 27–29]. In terminal urine, only 5% to 10% of filtered uric acid is excreted under normal conditions [25]. Net renal excretion of uric acid is determined by the balance among filtration, reabsorption and secretion along the nephron [1, 24, 26]. Besides renal elimination, approximately 30% of uric acid is excreted by the intestine, which has not been investigated in detail.

The kidney also performs a crucial role in glucose homeostasis. Similar to circulating uric acid, plasma glucose is freely filtered at the glomerulus, with almost all of it reabsorbed in the proximal tubule in normal situations [30]. As both glucose and uric acid are reabsorbed in the proximal tubule, glucose may influence renal uric acid excretion by regulation of uric acid reabsorption [15]. Most of filtered glucose (80–90%) is reabsorbed by the Sodium-glucose cotransporter 2 (SGLT2) in the S1 segment of the proximal tubule, while the Sodium-glucose cotransporter 1 (SGLT1) reabsorbs the remaining 10–20% in the more distal S2/S3 segment [31, 32]. SGLT2 inhibitors are a class of hypoglycemic drugs that inhibit the reabsorption of glucose in the proximal tubule by blocking SGLT2 [33, 34]. Interestingly, decreased SUA concentrations have been observed in subjects taking SGLT2 inhibitors in multiple randomized clinical trials [35–38], indicating a connection between glucose and uric acid metabolism in the kidney.

Glucose transporter protein-9 (GLUT9), expressed in human kidney proximal tubules, is a distinct member of the glucose transporters (GLUT) family due to its capacity for uric acid transportation. It is independent of sodium, chloride and anions, but is voltage dependent. GLUT9 shares common structural features with GLUT members such as 12 transmembrane helices, cytoplasmic amino and carboxytermini and an N-linked glycosylation site [39]. Using heterologous expression of GLUT9 in Xenopus oocytes, GLUT9 appears to be a functional transporter with low affinity for deoxyglucose [40]. Caulfied *et al*. also clarified that SLC2A9a, a splice variant of GLUT9, could exchange extracellular glucose for intracellular uric acid. These data suggest that glucose might influence the function of GLUT9 [41]. Therefore, it is assumed that an increase of glucose in tubular fluid with an associated elevation of reabsorptive transport on GLUT9 may inhibit uric acid reabsorption.

In the ascending segment, increased levels of insulin induced by elevated blood glucose levels may contribute to the reabsorption of uric acid. Consistent with previous studies [42], a positive correlation was found between FPG and serum insulin. Increased fasting serum insulin levels represent a compensatory mechanism to overcome insulin resistance [43]. After adjusting for insulin levels, the association between FPG and SUA is moderately attenuated, which suggests that the association is partly mediated through insulin. Previous studies also support this view. Using insulin clamp technique, Galvan *et al*. found that euglycemic hyperinsulinemia could cause correlated declines in fractional renal excretion of uric acid and sodium [44] and Maaten *et al*. showed similar results [45]. Thus, it is hypothesized that insulin could activate renal uric reabsorption through sodium-dependent anion transporters in brush-border membranes of the renal proximal tubule [4], since evidence for a direct effect of insulin on natriuresis has obtained both in normal subjects and in patients with essential hypertension [46]. In addition, high serum insulin levels may enhance renal uric acid reabsorption through elevated expression of the urate transporter-1, which is a urate exchanger mediating urate movement from urine to epithelium [47, 48]. Furthermore, increase in insulin levels could promote xanthine dehydrogenase and purine nucleoside phosphorylase, which participates in uric acid synthesis and elevates the level of uric acid in serum [49].

Our study has several limitations. The cross-sectional design precluded a definitive conclusion about causality. Fluctuations of circulating glucose and uric acid levels reveal modifications in tissue metabolism. Experiments on animal models and longitudinal studies with repeated measurements of plasma glucose and SUA would lend greater validity to our findings. Additionally, information on urinary uric acid clearance was not available, limiting further exploration of the association between FPG and SUA.

In summary, we report a U-shaped relationship between FPG and SUA concentrations in individuals with normal glucose tolerance. The association is partly mediated through serum insulin levels. Further studies with longitudinal designs would better reveal the underlying mechanisms for the relationship.

## Supporting information

S1 File Clinical datasets. The datasets supporting the conclusions of this article.
(XLSX)

## References

[1] So A, Thorens B. Uric acid transport and disease. The Journal of clinical investigation. 2010;120(6):1791–9. Epub 2010/06/03. doi: 10.1172/JCI42344 ; PubMed Central PMCID: PMCPmc2877959. 20516647 10.1172/JCI42344 PMC2877959

[2] Mount DB, Kwon CY, Zandi-Nejad K. Renal urate transport. Rheumatic diseases clinics of North America. 2006;32(2):313–31, vi. Epub 2006/05/24. doi: 10.1016/j.rdc.2006.02.006 . 16716882 10.1016/j.rdc.2006.02.006

[3] Maiuolo J, Oppedisano F, Gratteri S, Muscoli C, Mollace V. Regulation of uric acid metabolism and excretion. International journal of cardiology. 2016;213:8–14. Epub 2015/09/01. doi: 10.1016/j.ijcard.2015.08.109 . 26316329 10.1016/j.ijcard.2015.08.109

[4] Choi HK, Mount DB, Reginato AM. Pathogenesis of Gout. Annals of Internal Medicine. 2005;143(7):499–516. . 16204163 10.7326/0003-4819-143-7-200510040-00009

[5] Mandal AK, Mount DB. The molecular physiology of uric acid homeostasis. Annual review of physiology. 2015;77:323–45. Epub 2014/11/26. doi: 10.1146/annurev-physiol-021113-170343 . 25422986 10.1146/annurev-physiol-021113-170343

[6] Cicero AF, Derosa G, Rosticci M, D'Addato S, Agnoletti D, Borghi C. Long-term predictors of impaired fasting glucose and type 2 diabetes in subjects with family history of type 2 diabetes: a 12-years follow-up of the Brisighella Heart Study historical cohort. Diabetes research and clinical practice. 2014;104(1):183–8. Epub 2014/03/04. doi: 10.1016/j.diabres.2014.02.005 . 24582152 10.1016/j.diabres.2014.02.005

[7] Cicero AF, Rosticci M, Bove M, Fogacci F, Giovannini M, Urso R, et al Serum uric acid change and modification of blood pressure and fasting plasma glucose in an overall healthy population sample: data from the Brisighella heart study. Annals of medicine. 2017;49(4):275–82. Epub 2016/08/09. doi: 10.1080/07853890.2016.1222451 . 27499431 10.1080/07853890.2016.1222451

[8] Su J, Wei Y, Liu M, Liu T, Li J, Ji Y, et al Anti-hyperuricemic and nephroprotective effects of Rhizoma Dioscoreae septemlobae extracts and its main component dioscin via regulation of mOAT1, mURAT1 and mOCT2 in hypertensive mice. Archives of pharmacal research. 2014;37(10):1336–44. Epub 2014/05/29. doi: 10.1007/s12272-014-0413-6 . 24866061 10.1007/s12272-014-0413-6

[9] Kodama S, Saito K, Yachi Y, Asumi M, Sugawara A, Totsuka K, et al Association between serum uric acid and development of type 2 diabetes. Diabetes care. 2009;32(9):1737–42. Epub 2009/06/25. doi: 10.2337/dc09-0288 ; PubMed Central PMCID: PMCPmc2732137. 19549729 10.2337/dc09-0288 PMC2732137

[10] Liu Y, Jin C, Xing A, Liu X, Chen S, Li D, et al Serum uric acid levels and the risk of impaired fasting glucose: a prospective study in adults of north China. PloS one. 2013;8(12):e84712 Epub 2014/01/01. doi: 10.1371/journal.pone.0084712 ; PubMed Central PMCID: PMCPmc3871632. 24376838 10.1371/journal.pone.0084712 PMC3871632

[11] Herman JB, Goldbourt U. Uric acid and diabetes: observations in a population study. Lancet (London, England). 1982;2(8292):240–3. Epub 1982/07/31. . 6124672 10.1016/s0140-6736(82)90324-5

[12] Cook DG, Shaper AG, Thelle DS, Whitehead TP. Serum uric acid, serum glucose and diabetes: relationships in a population study. Postgraduate medical journal. 1986;62(733):1001–6. Epub 1986/11/01. ; PubMed Central PMCID: PMCPmc2418956. 3628142 10.1136/pgmj.62.733.1001 PMC2418956

[13] Tuomilehto J, Zimmet P, Wolf E, Taylor R, Ram P, King H. Plasma uric acid level and its association with diabetes mellitus and some biologic parameters in a biracial population of Fiji. American journal of epidemiology. 1988;127(2):321–36. Epub 1988/02/01. . 3337086 10.1093/oxfordjournals.aje.a114807

[14] Whitehead TP, Jungner I, Robinson D, Kolar W, Pearl A, Hale A. Serum urate, serum glucose and diabetes. Annals of clinical biochemistry. 1992;29 (Pt 2):159–61. Epub 1992/03/01. doi: 10.1177/000456329202900206 . 1626918 10.1177/000456329202900206

[15] Nan H, Dong Y, Gao W, Tuomilehto J, Qiao Q. Diabetes associated with a low serum uric acid level in a general Chinese population. Diabetes research and clinical practice. 2007;76(1):68–74. Epub 2006/09/12. doi: 10.1016/j.diabres.2006.07.022 . 16963150 10.1016/j.diabres.2006.07.022

[16] Oda E, Kawai R, Sukumaran V, Watanabe K. Uric acid is positively associated with metabolic syndrome but negatively associated with diabetes in Japanese men. Internal medicine (Tokyo, Japan). 2009;48(20):1785–91. Epub 2009/10/17. . 19834269 10.2169/internalmedicine.48.2426

[17] Hairong N, Zengchang P, Shaojie W, Weiguo G, Lei Z, Jie R, et al Serum uric acid, plasma glucose and diabetes. Diabetes & vascular disease research. 2010;7(1):40–6. Epub 2010/04/07. doi: 10.1177/1479164109347408 . 20368231 10.1177/1479164109347408

[18] Meisinger C, Doring A, Stockl D, Thorand B, Kowall B, Rathmann W. Uric acid is more strongly associated with impaired glucose regulation in women than in men from the general population: the KORA F4-Study. PloS one. 2012;7(5):e37180 Epub 2012/05/23. doi: 10.1371/journal.pone.0037180 ; PubMed Central PMCID: PMCPmc3353894. 22615932 10.1371/journal.pone.0037180 PMC3353894

[19] Li H, Zha X, Zhu Y, Liu M, Guo R, Wen Y. An Invert U-Shaped Curve: Relationship Between Fasting Plasma Glucose and Serum Uric Acid Concentration in a Large Health Check-Up Population in China. Medicine. 2016;95(16):e3456 Epub 2016/04/23. doi: 10.1097/MD.0000000000003456 ; PubMed Central PMCID: PMCPmc4845851. 27100447 10.1097/MD.0000000000003456 PMC4845851

[20] Levey AS, Stevens LA, Schmid CH, Zhang Y, Castro AF, Feldman HI, et al A New Equation to Estimate Glomerular Filtration Rate. Annals of Internal Medicine. 2009;150(9):604–12. . 19414839 10.7326/0003-4819-150-9-200905050-00006 PMC2763564

[21] Matthews DR, Hosker JP, Rudenski AS, Naylor BA, Treacher DF, Turner RC. Homeostasis model assessment: insulin resistance and beta-cell function from fasting plasma glucose and insulin concentrations in man. Diabetologia. 1985;28(7):412–9. Epub 1985/07/01. . 3899825 10.1007/BF00280883

[22] Andersoncook CM. Generalized Additive Models: An Introduction With R. Simon N. Wood. Journal of the American Statistical Association. 2007;102:760–1.

[23] Chow GC. TESTS OF EQUALITY BETWEEN SETS OF COEFFICIENTS IN TWO LINEAR REGRESSIONS. Econometrica. 1960;28(3):591–605.

[24] Maesaka JK, Fishbane S. Regulation of renal urate excretion: a critical review. American journal of kidney diseases: the official journal of the National Kidney Foundation. 1998;32(6):917–33. Epub 1998/12/18. . 9856507 10.1016/s0272-6386(98)70067-8

[25] Lipkowitz MS. Regulation of uric acid excretion by the kidney. Current rheumatology reports. 2012;14(2):179–88. Epub 2012/02/24. doi: 10.1007/s11926-012-0240-z . 22359229 10.1007/s11926-012-0240-z

[26] Levinson DJ, Sorensen LB. Renal handling of uric acid in normal and gouty subject: evidence for a 4-component system. Annals of the rheumatic diseases. 1980;39(2):173–9. Epub 1980/04/01. ; PubMed Central PMCID: PMCPmc1000505. 7387222 10.1136/ard.39.2.173 PMC1000505

[27] Podevin R, Ardaillou R, Paillard F, Fontanelle J, Richet G. [Study in man of the kinetics of the appearance of uric acid 2-14C in the urine]. Nephron. 1968;5(2):134–40. Epub 1968/01/01. . 5655614 10.1159/000179623

[28] Abramson RG, Levitt MF. Use of pyrazinamide to assess renal uric acid transport in the rat: a micropuncture study. The American journal of physiology. 1976;230(5):1276–83. Epub 1976/05/01. . 1275069 10.1152/ajplegacy.1976.230.5.1276

[29] Chaudhary K, Malhotra K, Sowers J, Aroor A. Uric Acid—key ingredient in the recipe for cardiorenal metabolic syndrome. Cardiorenal medicine. 2013;3(3):208–20. Epub 2014/01/24. doi: 10.1159/000355405 ; PubMed Central PMCID: PMCPmc3884201. 24454316 10.1159/000355405 PMC3884201

[30] Mather A, Pollock C. Glucose handling by the kidney. Kidney international Supplement. 2011;(120):S1–6. Epub 2011/03/05. doi: 10.1038/ki.2010.509 . 21358696 10.1038/ki.2010.509

[31] Wright EM, Loo DD, Hirayama BA. Biology of human sodium glucose transporters. Physiological reviews. 2011;91(2):733–94. Epub 2011/04/30. doi: 10.1152/physrev.00055.2009 . 21527736 10.1152/physrev.00055.2009

[32] Vrhovac I, Balen Eror D, Klessen D, Burger C, Breljak D, Kraus O, et al Localizations of Na(+)-D-glucose cotransporters SGLT1 and SGLT2 in human kidney and of SGLT1 in human small intestine, liver, lung, and heart. Pflugers Archiv: European journal of physiology. 2015;467(9):1881–98. Epub 2014/10/12. doi: 10.1007/s00424-014-1619-7 . 25304002 10.1007/s00424-014-1619-7

[33] Nair S, Wilding JP. Sodium glucose cotransporter 2 inhibitors as a new treatment for diabetes mellitus. The Journal of clinical endocrinology and metabolism. 2010;95(1):34–42. Epub 2009/11/07. doi: 10.1210/jc.2009-0473 . 19892839 10.1210/jc.2009-0473

[34] Bailey CJ. Renal glucose reabsorption inhibitors to treat diabetes. Trends in pharmacological sciences. 2011;32(2):63–71. Epub 2011/01/08. doi: 10.1016/j.tips.2010.11.011 . 21211857 10.1016/j.tips.2010.11.011

[35] Bailey CJ, Gross JL, Pieters A, Bastien A, List JF. Effect of dapagliflozin in patients with type 2 diabetes who have inadequate glycaemic control with metformin: a randomised, double-blind, placebo-controlled trial. Lancet (London, England). 2010;375(9733):2223–33. Epub 2010/07/09. doi: 10.1016/s0140-6736(10)60407-2 . 20609968 10.1016/S0140-6736(10)60407-2

[36] Cefalu WT, Leiter LA, Yoon KH, Arias P, Niskanen L, Xie J, et al Efficacy and safety of canagliflozin versus glimepiride in patients with type 2 diabetes inadequately controlled with metformin (CANTATA-SU): 52 week results from a randomised, double-blind, phase 3 non-inferiority trial. Lancet (London, England). 2013;382(9896):941–50. Epub 2013/07/16. doi: 10.1016/s0140-6736(13)60683-2 . 23850055 10.1016/S0140-6736(13)60683-2

[37] Wilding JP, Ferrannini E, Fonseca VA, Wilpshaar W, Dhanjal P, Houzer A. Efficacy and safety of ipragliflozin in patients with type 2 diabetes inadequately controlled on metformin: a dose-finding study. Diabetes, obesity & metabolism. 2013;15(5):403–9. Epub 2012/11/21. doi: 10.1111/dom.12038 . 23163880 10.1111/dom.12038

[38] Ferrannini E, Seman L, Seewaldt-Becker E, Hantel S, Pinnetti S, Woerle HJ. A Phase IIb, randomized, placebo-controlled study of the SGLT2 inhibitor empagliflozin in patients with type 2 diabetes. Diabetes, obesity & metabolism. 2013;15(8):721–8. Epub 2013/02/13. doi: 10.1111/dom.12081 . 23398530 10.1111/dom.12081

[39] Augustin R, Carayannopoulos MO, Dowd LO, Phay JE, Moley JF, Moley KH. Identification and characterization of human glucose transporter-like protein-9 (GLUT9): alternative splicing alters trafficking. The Journal of biological chemistry. 2004;279(16):16229–36. Epub 2004/01/24. doi: 10.1074/jbc.M312226200 . 14739288 10.1074/jbc.M312226200

[40] Clemencon B, Luscher BP, Fine M, Baumann MU, Surbek DV, Bonny O, et al Expression, purification, and structural insights for the human uric acid transporter, GLUT9, using the Xenopus laevis oocytes system. PloS one. 2014;9(10):e108852 Epub 2014/10/07. doi: 10.1371/journal.pone.0108852 ; PubMed Central PMCID: PMCPmc4186817. 25286413 10.1371/journal.pone.0108852 PMC4186817

[41] Caulfield MJ, Munroe PB, O'Neill D, Witkowska K, Charchar FJ, Doblado M, et al SLC2A9 is a high-capacity urate transporter in humans. PLoS medicine. 2008;5(10):e197 Epub 2008/10/10. doi: 10.1371/journal.pmed.0050197 ; PubMed Central PMCID: PMCPmc2561076. 18842065 10.1371/journal.pmed.0050197 PMC2561076

[42] Aswani R, Lochow A, Dementieva Y, Lund VA, Elitsur Y. Acanthosis nigricans as a clinical marker to detect insulin resistance in Caucasian children from West Virginia. Clinical pediatrics. 2011;50(11):1057–61. Epub 2011/07/16. doi: 10.1177/0009922811414288 . 21757774 10.1177/0009922811414288

[43] Olefsky J, Farquhar JW, Reaven G. Relationship between fasting plasma insulin level and resistance to insulin-mediated glucose uptake in normal and diabetic subjects. Diabetes. 1973;22(7):507–13. Epub 1973/07/01. . 4719190 10.2337/diab.22.7.507

[44] Quinones Galvan A, Natali A, Baldi S, Frascerra S, Sanna G, Ciociaro D, et al Effect of insulin on uric acid excretion in humans. The American journal of physiology. 1995;268(1 Pt 1):E1–5. Epub 1995/01/01. . 7840165 10.1152/ajpendo.1995.268.1.E1

[45] Ter Maaten JC, Voorburg A, Heine RJ, Ter Wee PM, Donker AJ, Gans RO. Renal handling of urate and sodium during acute physiological hyperinsulinaemia in healthy subjects. Clinical science (London, England: 1979). 1997;92(1):51–8. Epub 1997/01/01. . 9038591 10.1042/cs0920051

[46] Muscelli E, Natali A, Bianchi S, Bigazzi R, Galvan AQ, Sironi AM, et al Effect of insulin on renal sodium and uric acid handling in essential hypertension. American journal of hypertension. 1996;9(8):746–52. Epub 1996/08/01. . 8862220 10.1016/0895-7061(96)00098-2

[47] Enomoto A, Kimura H, Chairoungdua A, Shigeta Y, Jutabha P, Cha SH, et al Molecular identification of a renal urate anion exchanger that regulates blood urate levels. Nature. 2002;417(6887):447–52. Epub 2002/05/25. doi: 10.1038/nature742 . 12024214 10.1038/nature742

[48] Doshi M, Takiue Y, Saito H, Hosoyamada M. The increased protein level of URAT1 was observed in obesity/metabolic syndrome model mice. Nucleosides, nucleotides & nucleic acids. 2011;30(12):1290–4. Epub 2011/12/03. doi: 10.1080/15257770.2011.603711 . 22132989 10.1080/15257770.2011.603711

[49] Wu JM, Nickels JS, Fisher JR. Regulation of nitrogen catabolic enzymes in chick liver: effects of insulin. Enzyme. 1977;22(1):60–9. Epub 1977/01/01. . 13991 10.1159/000458509

